# STATH Downregulation and Poor Prognosis in Head and Neck Squamous Cell Carcinoma: Transcriptomic Analysis Reveals Poor Impact on Oral Health

**DOI:** 10.7759/cureus.99357

**Published:** 2025-12-16

**Authors:** Aisha Elnagi, Einas Elgassim, Fadwa Mahjoub, Afraa Elmahdi, Baraah Ahmed, Mohamed Alfaki

**Affiliations:** 1 Dentistry, Independent Researcher, Doha, QAT; 2 Medicine, Weill Cornell Medicine - Qatar, Doha, QAT; 3 Dentistry, Independent Researcher, Lausanne, CHE; 4 Dentistry, Sudan University of Science and Technology, Khartoum, SDN; 5 Medicine, Independent Researcher, Buraydah, SAU; 6 Computer Science, Al-Neelain University, Khartoum, SDN

**Keywords:** bioinformatics, diagnostic biomarker, gene expression, head and neck cancer, oral health, prognostic biomarker, salivary diagnostics, squamous cell carcinoma, stath, statherin

## Abstract

Introduction

Salivary statherin, encoded by the STATH gene, maintains enamel integrity and oral microbial balance. Its downregulation has been linked to oral diseases, but its transcriptomic behavior in head and neck squamous cell carcinoma (HNSCC) remains largely unexplored. This study aimed to assess STATH as a diagnostic and prognostic biomarker in HNSCC and explore its potential biological role.

Materials and methods

The expression of STATH across 33 cancer types was analyzed via TIMER (Tumor Immune Estimation Resource), GEPIA (Gene Expression Profiling Interactive Analysis), and UALCAN (University of Alabama at Birmingham Cancer Data Analysis Portal), and promoter methylation and clinical correlations were explored in HNSCC. Prognostic significance was evaluated via Kaplan-Meier survival analysis (log-rank p<0.05). Immune cell infiltration was assessed by human papilloma virus (HPV) status via TIMER. Mutation data (n=674) were analyzed via cBioPortal. The findings were validated via the GEO GSE6631 dataset (n=22 paired samples). Co-expression and pathway enrichment analyses were conducted via UALCAN and Enrichr. Protein-protein interaction (PPI) networks were constructed via Search Tool for the Retrieval of Interacting Genes/Proteins (STRING) database.

Results

STATH was significantly downregulated in HNSCC tissues compared with normal tissues according to TIMER (p<0.001) and GEPIA (p<0.01), with slightly reduced promoter methylation (median β=0.611; p=0.006) and rare genetic alterations (<1%). Higher STATH expression was associated with better overall survival (hazard ratio (HR)=0.914; log-rank p=0.033), especially in HPV-positive patients (HR=0.822; log-rank p=0.039). STATH expression was correlated with immune infiltration, particularly B cells, in HPV-positive tumors (r=0.355; p=0.001). STATH downregulation was validated (adjusted p=0.004). Co-expression analysis revealed enrichment in immune and oral health-associated pathways, including salivary protein's role in periodontitis (p=0.007; OR=118.8; LYZ, LTF) and dental caries (p=0.007; OR=59.36; LYZ, CCL28). PPIs interact with histatins and mucins.

Conclusion

STATH is consistently downregulated in HNSCC, independent of promoter methylation or genetic mutations. Its suppression likely reflects tumor-driven remodeling of the salivary microenvironment, weakening host defenses and promoting disease progression. STATH has potential as a noninvasive diagnostic and prognostic biomarker.

## Introduction

Head and neck squamous cell carcinoma (HNSCC) is the seventh most common cancer worldwide, with 890,000 new cases and 450,000 deaths annually [1]. Poor oral health has been linked to the survival of HNSCC patients [2]. Up to 74.9% of HNSCC patients present with periodontal disease, caries, or tooth loss prior to treatment [3]. HNSCC patients frequently display gingival inflammation and overall poorer oral health [4]. Epidemiological studies have shown that periodontal disease and tooth loss significantly increase the risk of HNSCC [5].

Given the proximity of head and neck tumors to the salivary glands, saliva represents a potentially informative diagnostic biofluid. It contains a variety of protective proteins, including immunoglobulins, cystatins, histatins, lactoferrin, mucins, and statherin, which together help maintain oral health [6]. Among these proteins, statherin, encoded by the STATH gene, is particularly unique. It regulates enamel homeostasis, prevents mineral precipitation, and provides antimicrobial defense [7]. As a major component of the acquired enamel pellicle (AEP), statherin protects against acid-induced demineralization, uniquely inhibits spontaneous calcium phosphate precipitation, and modulates hydroxyapatite crystal formation on the tooth surface, which together limits calculus formation [8].

Studies have shown that low statherin levels are associated with poor oral health and are correlated with increased dental caries severity and microbial imbalance [9,10]. Other studies have identified statherin as a promising biomarker for caries [11]. Despite interest in the use of statherin as a salivary biomarker, the transcriptomic regulation of the STATH gene in HNSCC remains largely unexplored, representing a gap that this study aims to address.

Although the physiological role of STATH in saliva is well established, its function in cancer biology remains unclear, and no definitive conclusions have been reached. In line with the hallmark of reprogramming of the tumor microenvironment, we hypothesize that HNSCC suppresses STATH expression to remodel the salivary microenvironment, weaken host defense, and promote tumor-supportive inflammation. Given that STATH is abundantly present in saliva and can be reliably detected in biological samples [12], its downregulation in HNSCC may serve as a specific and noninvasive diagnostic biomarker. We also propose that HNSCC tumors actively reduce STATH expression, altering the salivary microenvironment in a way that impairs host defense and promotes poor oral health, potentially creating a feedback loop that remodels the tumor microenvironment to favor HNSCC progression.

Therefore, this study aimed to assess STATH as a diagnostic and prognostic biomarker in HNSCC by analyzing its expression in tumor tissues compared with normal tissues, and to explore its potential biological role.

## Materials and methods

Design and setting of the study

This is an in silico study that utilized publicly available transcriptomic datasets from online databases.

Assessing diagnostic potential

To investigate the potential of STATH as a diagnostic biomarker in HNSCC, we analyzed its expression profile across 33 cancer types via the following public databases: TIMER (Tumor Immune Estimation Resource), GEPIA (Gene Expression Profiling Interactive Analysis), and UALCAN (University of Alabama at Birmingham Cancer Data Analysis Portal) [13-15]. STATH expression levels were compared between tumor and normal tissue samples. A cancer type was considered for further analysis only if STATH showed consistent and statistically significant dysregulation (p<0.05, 0.01, or 0.001, depending on the database) in at least two of the three reported databases. On the basis of our selection criteria, HNSCC was the only cancer type demonstrating consistent and significant dysregulation of STATH.

To investigate STATH expression across different HNSCC stages, we generated violin plots using GEPIA (Gene Expression Profiling Interactive Analysis). UALCAN was employed to examine the differential expression of STATH across different clinical parameters and subgroups and to analyze promoter DNA methylation levels, as well as across different clinical parameters (p<0.05). Beta values (β), ranging from 0 (unmethylated) to 1 (fully methylated), were used to quantify DNA methylation. β value cutoffs were considered to indicate hypermethylation (0.5-0.7) or hypomethylation (0.25-0.3). Median β values were compared between tumor and normal samples (p<0.01). Box plots showing STATH expression in tumor and normal samples were generated via built-in visualization tools from TIMER, GEPIA, and UALCAN.

Assessing prognostic potential

Correlations with immune cell infiltration were evaluated in the TIMER database only. The abundances of the following six immune cells were evaluated: B cells, CD4+ T cells, CD8+ T cells, macrophages, neutrophils, and dendritic cells. Correlations were assessed with Spearman’s correlation coefficient, with p<0.05 indicating statistical significance. The findings were visualized as scatter plots via TIMERS built-in tools. Correlation strength was classified as negative or positive: very weak (0-0.1), weak (0.1-0.3), moderate (0.3-0.5), strong (0.5-0.7), or very strong (0.7-1.0). Overall survival (OS) was analyzed via TIMER, GEPIA, UACLAN, and Kaplan-Meier plot [13-16]. A log-rank test was used to assess whether there was a difference in survival time between the high and low groups, and p<0.05 was considered statistically significant. Hazard ratio (HR)>1 was interpreted as having a higher risk of death for the low-expression group (poorer prognosis), whereas an HR<1 indicated a lower risk of death (better prognosis). The results were assembled into a spreadsheet to identify findings that were consistent and statistically significant across at least two databases. Kaplan-Meier plots were generated via built-in tools in each database.

Mutation analysis

We further analyzed genomic alterations/mutations in the STATH gene via cBioPortal for Cancer Genomics [17]. Data were obtained from four HNSCC studies, with a total of 674 samples: oral squamous cell carcinoma (MD Anderson, Cancer Discov 2013), head and neck squamous cell carcinoma (Johns Hopkins, Science 2011), head and neck squamous cell carcinoma (Broad, Science 2011), and head and neck squamous cell carcinoma (TCGA, Firehose Legacy). We examined the mutation frequency of STATH and assessed its impact on overall survival, which was visualized via Kaplan-Meier curves. A log-rank p value <0.05 was considered statistically significant. Using built-in visualization tools, an OncoPrint was generated to visualize the types and distributions of mutations, and a bar chart was used to display mutation types and frequencies.

Validation of results using the GEO dataset

To confirm and validate our results, we initially queried the Gene Expression Omnibus (GEO) for independent datasets containing HNSCC samples [18]. Dataset GSE6631, titled expression data from head and neck squamous cell carcinoma, was selected and analyzed via GEO2R [19]. This dataset included paired tumor and normal samples from 22 patients. The samples were defined as tumor or normal, and differential expression analysis was performed. An adjusted p value <0.05 was considered statistically significant, and a log2-fold change was used to assess the degree of STATH dysregulation. Volcano plots were generated via the ggplot2 package to visualize differential gene expression [20]. The cutoff criteria were set as a |log2fold change (FC)≥1| and adjusted p value <0.05. Genes meeting these criteria were considered significantly differentially expressed.

Co-expression and pathway enrichment analysis

A list of the top 25 genes most positively correlated with STATH in HNSCC was retrieved from UALCAN, which calculates Pearson correlation coefficients on the basis of The Cancer Genome Atlas (TCGA) expression data. Genes with very low expression (median TPM<0.5) were excluded. Genes with a correlation coefficient |r|≥0.3 and a significance threshold of p<0.05 were considered significantly correlated. To visualize the co-expression patterns, scatter plots and a heatmap of the top 25 positively correlated genes were obtained from UALCAN. To reveal the functional role of STATH in HNSCC tumorigenesis, the top 25 co-expressed genes were analyzed via the Enrichr *Homo sapiens* library, querying Elsevier for pathway enrichment [21], Gene Ontology Biological Process (BP), Gene Ontology Cellular Component (CC), and Gene Ontology Molecular Function (MF) for GO terms. To explore the potential biological functions of STATH, pathways and GO terms with adjusted p values <0.05 were considered significantly enriched.

Protein-protein interaction network construction

The protein-protein interaction (PPI) network was constructed via the Search Tool for the Retrieval of Interacting Genes/Proteins database (STRING) version 12.0 [22]. The analysis was performed via the full STRING network, with interaction edges on the basis of evidence and a high confidence threshold of 0.7, which is sometimes relaxed to a medium confidence threshold of 0.4 when few or no interactions are observed. PPI enrichment p values <0.05 were considered statistically significant. STATH was queried as a single protein to assess its general protein interactions, and the top 25 identified co-expressed genes were used to investigate its functional role in HNSCC. Hub genes were identified on the basis of node degree. The resulting network was visualized through the STRING interface to identify functionally related clusters and potential interaction partners of STATH.

## Results

Expression profile of STATH

**Figure 1:**
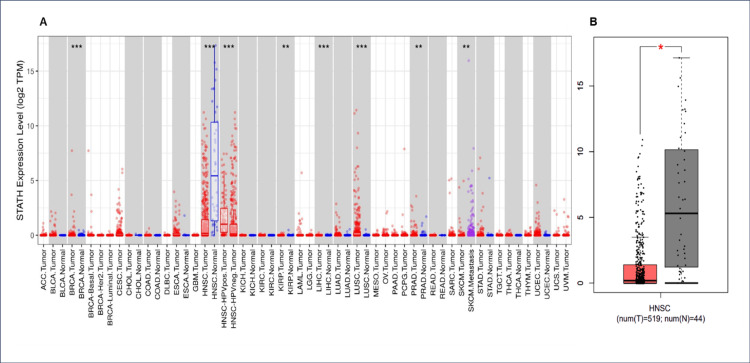
Expression analysis of the STATH gene in human cancers. A: STATH expression across different cancers in TIMER. B: Comparison of STATH expression between tumor and normal tissues via GEPIA. TIMER: Tumor Immune Estimation Resource; GEPIA: Gene Expression Profiling Interactive Analysis.

An assessment of STATH expression levels across 33 cancer types showed that STATH was significantly and consistently downregulated in HNSCC tumors compared with normal controls. This was the case for both the TIMER (p<0.001) and the GEPIA databases tumor samples (n=519) and normal samples (n=44) (p<0.01) (Figure 1). Moreover, UACLAN showed nonsignificant results. There were no significant differences in STATH expression across different stages of HNSCC (analysis of variance (ANOVA): F=0.11, p=0.954).

Clinical parameters in HNSCC

**Figure 2:**
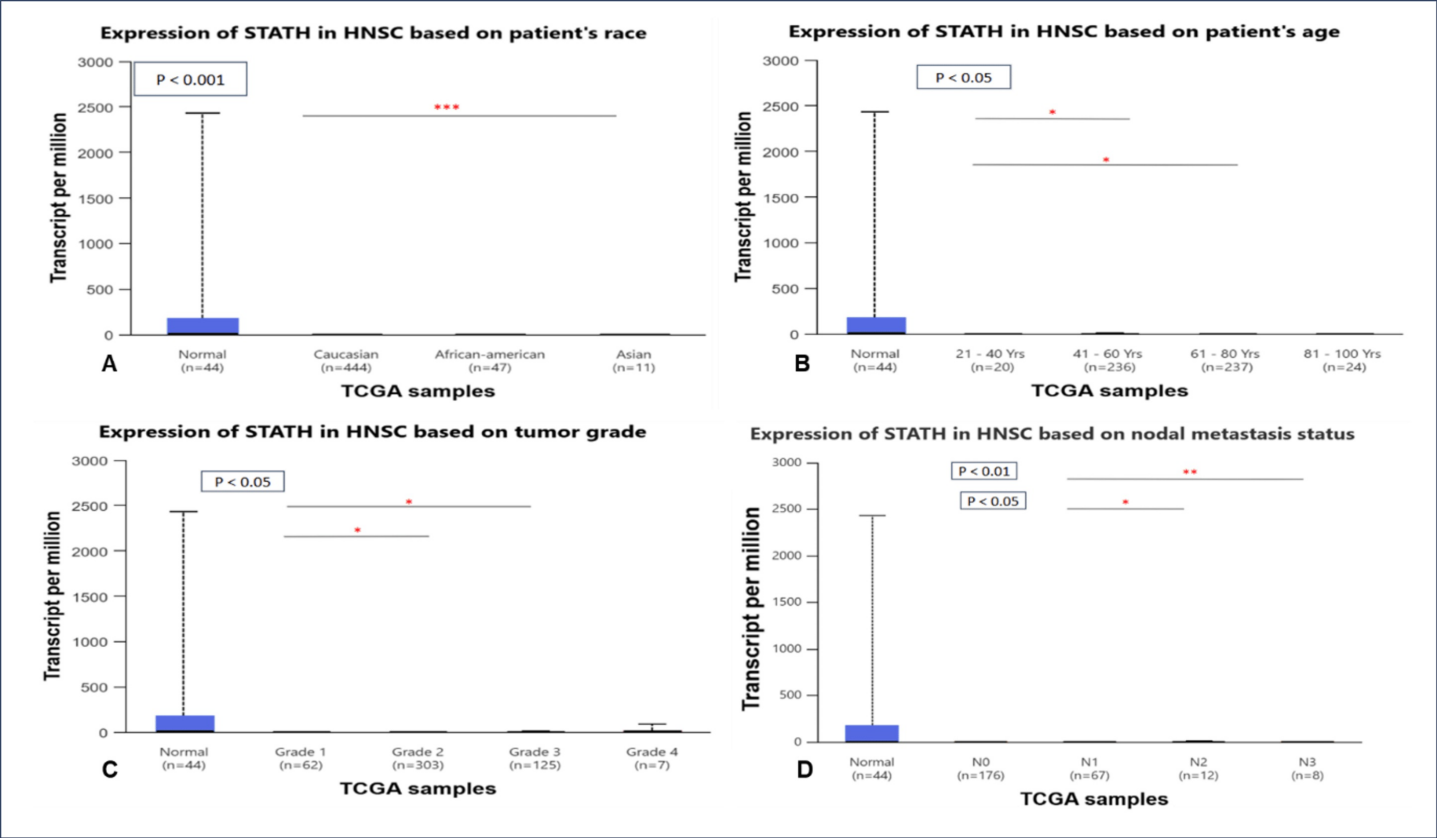
STATH gene expression in HNSCC across clinical parameters. A: STATH expression according to patient race. B: STATH expression according to patient age. C: STATH expression based on tumor grade. D: STATH expression based on nodal metastasis status. HNSCC: Head and neck squamous cell carcinoma

STATH expression was significantly lower in Asian patients than in Caucasian patients (p<0.001). However, no significant differences were detected among the other racial groups (Figure 2A). A comparison of the different age groups revealed that STATH was significantly downregulated in individuals aged 21-40 years compared with those aged 41-60 years (p=0.02) and 61-80 years (p=0.04) (Figure 2B). All other age-based comparisons were insignificant. In terms of tumor grade, significant differences were found between Grade 1 and Grade 2 tumors (p=0.03) and Grade 1 and Grade 3 tumors (p=0.04) (Figure 2C), with no significant differences between the other grades. In terms of nodal metastasis, STATH was significantly downregulated in N1 vs. N2 (p=0.03) and in N1 compared with N3 (p=0.007) (Figure 2D). Other nodal metastasis-based comparisons revealed no significant differences.

Promoter methylation profile of STATH in HNSCC

**Figure 3:**
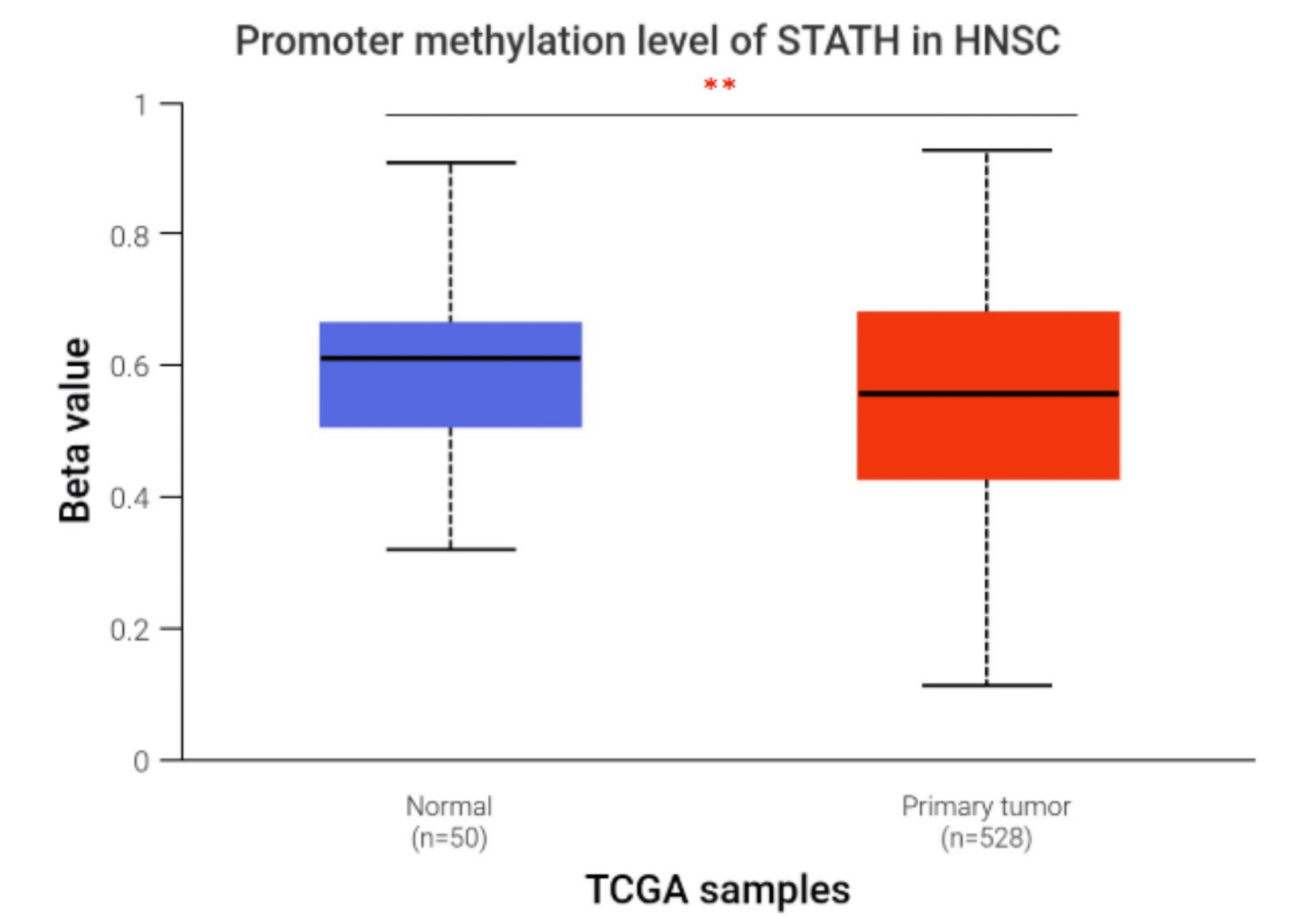
Promoter methylation of STATH in HNSCC compared with normal tissues.

Both normal and tumor tissues were hypermethylated. However, compared with normal tissues, tumor tissues presented a significant reduction in methylation (median β=0.611; p=0.006) (Figure 3).

Genetic alterations of STATH in HNSCC

**Figure 4:**
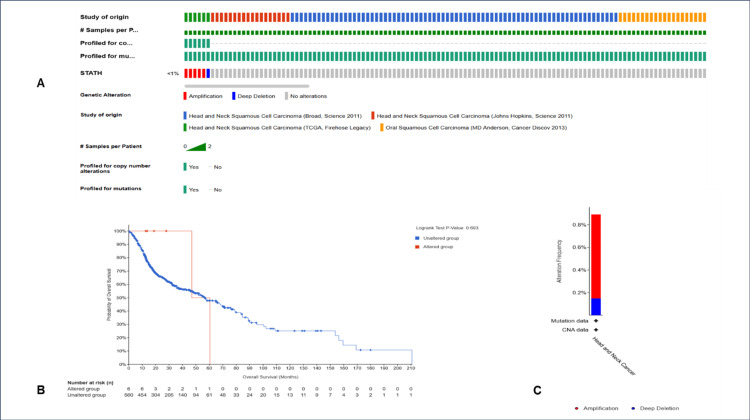
Mutation analysis of STATH in HNSCC. A. OncoPrint B. OS analysis between the altered and unaltered groups C. frequency of mutations in HNSCC studies. OS: Overall survival; HNSCC: head and neck squamous cell carcinoma.

A total of four studies and 676 samples were selected, and STATH was altered in six cases (<1%). The top mutation type was gene amplification, followed by deep deletion (Figure 4). The overall survival analysis revealed no significant difference between patients with and without STATH alterations (log-rank test, p=0.693; p>0.05).

Immune cell infiltration analysis of STATH in HNSCC

**Figure 5:**
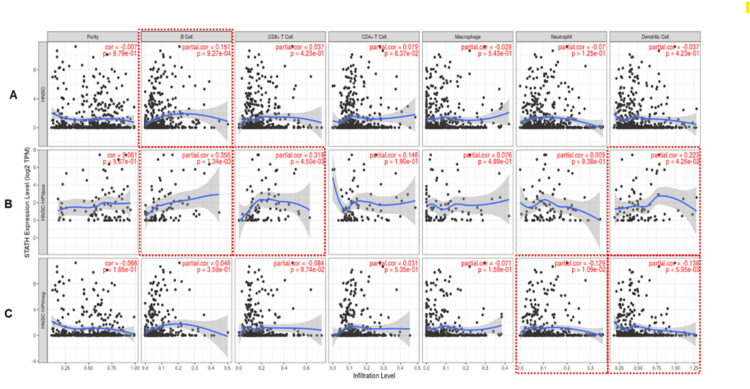
Immune cell infiltration analysis of STATH in HNSCC. A. HNSCC overall B. HPV-positive HNSCC C. HPV-negative HNSCC. HNSCC: Head and neck squamous cell carcinoma; HPV: human papilloma virus.

Overall, STATH expression was significantly correlated with B cells (partial cor=0.151, p<0.001) in HNSCC (Figure 5A). Further analysis revealed that the types of cells correlated with STATH varied on the basis of HPV status. In HPV-positive HNSCC, STATH expression was significantly correlated with B cells (partial cor=0.355, p=0.001), CD8⁺ T cells (partial cor=0.318, p=0.0045), and dendritic cells (partial cor=0.223, p=0.043). (Figure 4B). On the other hand, STATH expression in HPV-negative HNSCC was significantly negatively correlated with neutrophil infiltration (partial cor=-0.129, p=0.01) and dendritic cell infiltration (partial cor=-0.138, p=0.006) (Figure 5C).

Overall survival of HNSCC patients

**Figure 6:**
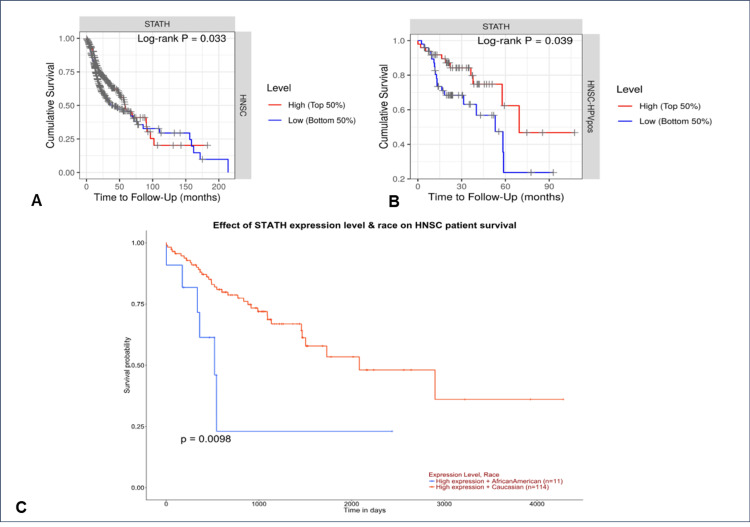
Overall survival analysis of STATH in HNSCC. A. Kaplan-Meier curve showing the overall survival curve. B. In HPV-positive tumors only. C. Overall survival of African Americans and Caucasian races. HNSCC: Head and neck squamous cell carcinoma

Higher STATH levels were significantly associated with better survival outcomes than lower STATH levels were (hazard ratio (HR)=0.914, log-rank p=0.033) (Figure 6A). Similarly, in HPV-positive HNSCC patients, high STATH expression was correlated with better overall survival than low STATH expression was (HR=0.822, log-rank p=0.039) (Figure 6B). Patients with high STATH expression in the Caucasian group had the most favorable overall survival, whereas those with high expression in the African American group had the least favorable survival (p=0.0098) (Figure 6C).

Validation of STATH expression in HNSCC

**Figure 7:**
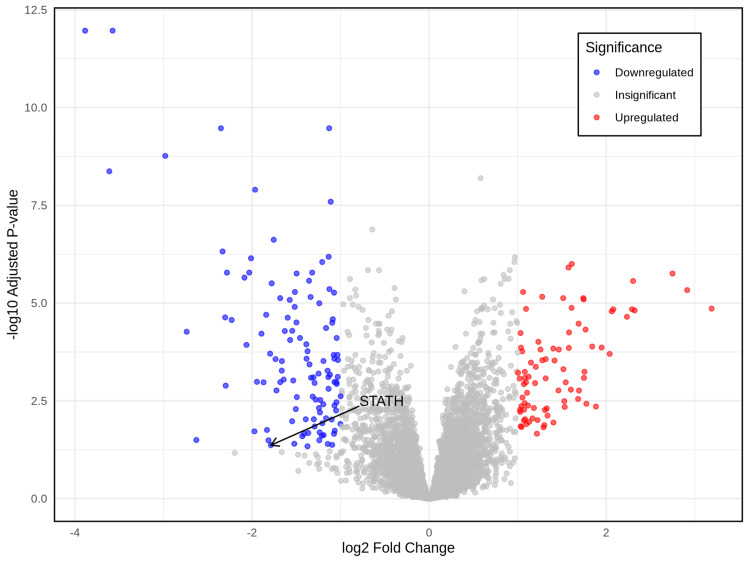
Volcano plot displaying the downregulation of STATH among differentially expressed genes in HNSCC. HNSCC: Head and neck squamous cell carcinoma.

Differential analysis of 22 paired tumor and normal samples revealed that STATH was significantly downregulated in tumor tissues compared with normal controls (adjusted p=0.004) (Figure 7).

Co-expression and pathway enrichment analysis

**Figure 8:**
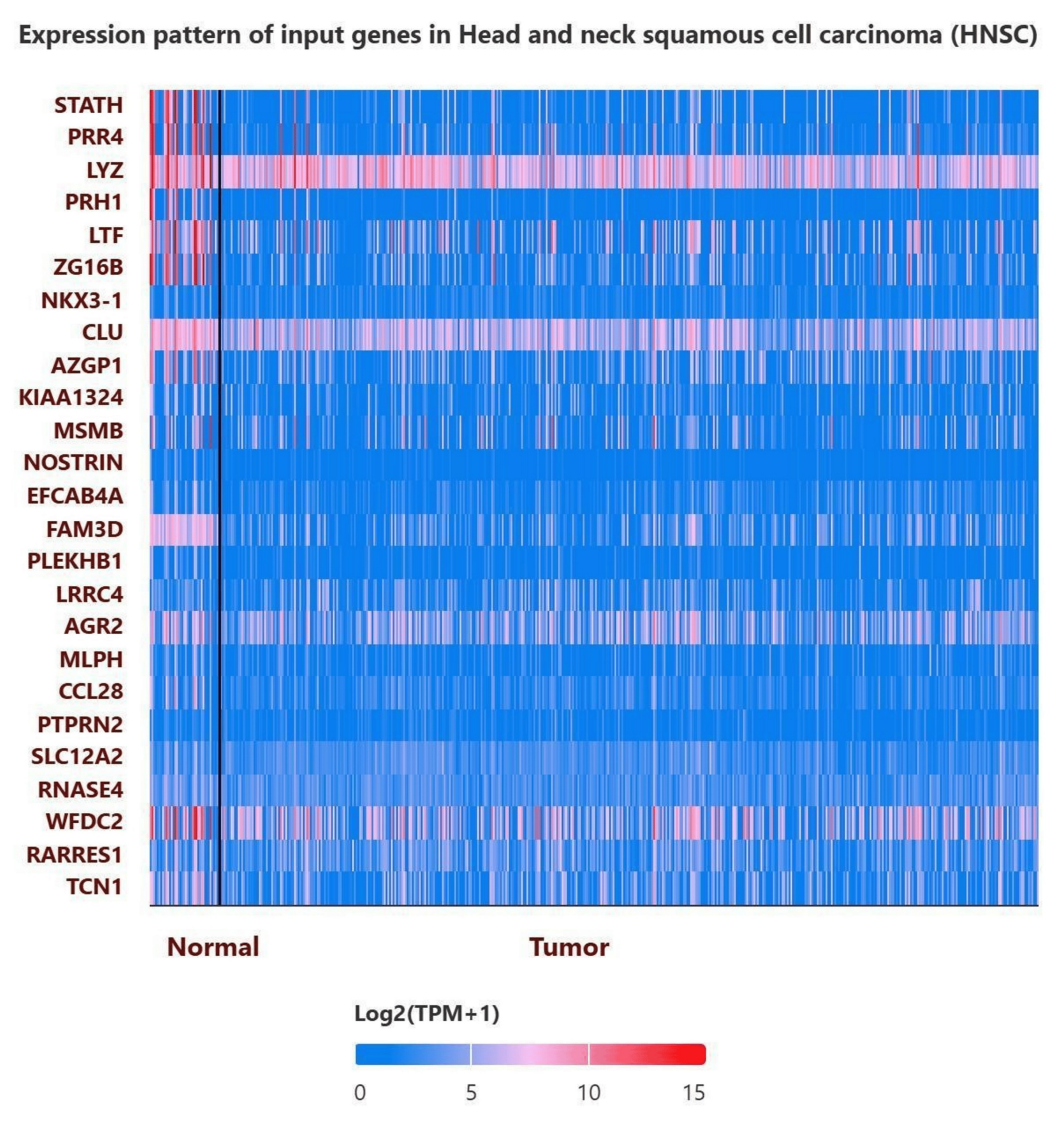
Expression patterns of the top 25 genes positively correlated with STATH expression in HNSCC. HNSCC: Head and neck squamous cell carcinoma.

The top 25 genes positively correlated with STATH in HNSCC are shown in Figure 8.

STATH co-expression analysis revealed significant enrichment in 10 GO terms (Table 1). The key associated pathways include proteins involved in periodontitis, chronic bronchitis, prostate cancer, dental caries, ulcerative colitis, presbycusis, neutrophil degranulation via FPR1 signaling and amyloid beta clearance in Alzheimer’s disease. These genes contribute to antimicrobial and antibacterial humoral responses, defense against bacteria and defense against gram-positive bacteria. Molecularly, endopeptidase inhibitor activity is significant. Cellularly, they are localized to their tertiary and specific granules. The strongest association based on odds ratios in the Elsevier pathway was in amyloid beta clearance in Alzheimer’s disease (odds ratio (OR)=199.7, p=0.04), with CLU being the only gene associated with this pathway. Other strongly associated proteins in this pathway were salivary proteins involved in periodontitis (OR=118.80, adjusted, p=0.007; genes: LYZ, LTF) and proteins involved in dental caries (OR=59.36, p=0.007; genes: LYZ, CCL28). 

**Table 1:** Gene Ontology and pathway enrichment of the top co-expressed genes associated with STATH in HNSCC. HNSCC: Head and neck squamous cell carcinoma.

GOTERM	Adjusted P value	Odds Ratio	Genes
GOTERM_BP			
Antimicrobial Humoral Response (GO:0019730)	0.0003	33.25	RNASE4;LYZ;CCL28;WFDC2;LTF
Antibacterial Humoral Response (GO:0019731)	0.006	51.97	RNASE4;WFDC2;LTF
Defense Response to Bacterium (GO:0042742)	0.02	16.55	RNASE4;LYZ;WFDC2;LTF
Defense Response to gram-positive Bacterium (GO:0050830)	0.02	27.29	RNASE4;LYZ;LTF
GOTERM_CC			
Tertiary Granule Lumen (GO:1904724)	0.001	50.95	TCN1;LYZ;LTF
Tertiary Granule (GO:0070820)	0.001	22.65	PTPRN2;TCN1;LYZ;LTF
Specific Granule Lumen (GO:0035580)	0.001	44.78	TCN1;LYZ;LTF
Secretory Granule Lumen (GO:0034774)	0.008	11.45	TCN1;LYZ;CLU;LTF
Specific Granule (GO:0042581)	0.01	16.57	TCN1;LYZ;LTF
GOTERM_MF			
Endopeptidase Inhibitor Activity (GO:0004866)	0.03	22.33	RARRES1;WFDC2;LTF
Elsevier pathway			
Salivary Proteins Role in Periodontitis	0.007	118.80	LYZ;LTF
Proteins Involved in Chronic Bronchitis	0.007	72.28	LYZ;LTF
Prostate Cancer	0.007	21.58	CLU;NKX3-1;MSMB
Proteins Involved in Dental Caries	0.007	59.36	LYZ;CCL28
Proteins Involved in Prostate Cancer	0.007	19.16	CLU;NKX3-1;MSMB
Proteins Involved in Ulcerative Colitis	0.007	18.74	TFF3;LYZ;LTF
Proteins Involved in Presbycusis	0.01	38.62	SLC12A2;TFF3
Neutrophil Degranulation via FPR1 Signaling	0.02	27.65	LYZ;LTF
Amyloid beta Clearance in Alzheimer Disease	0.04	199.7	CLU

Protein-protein interaction network

**Figure 9:**
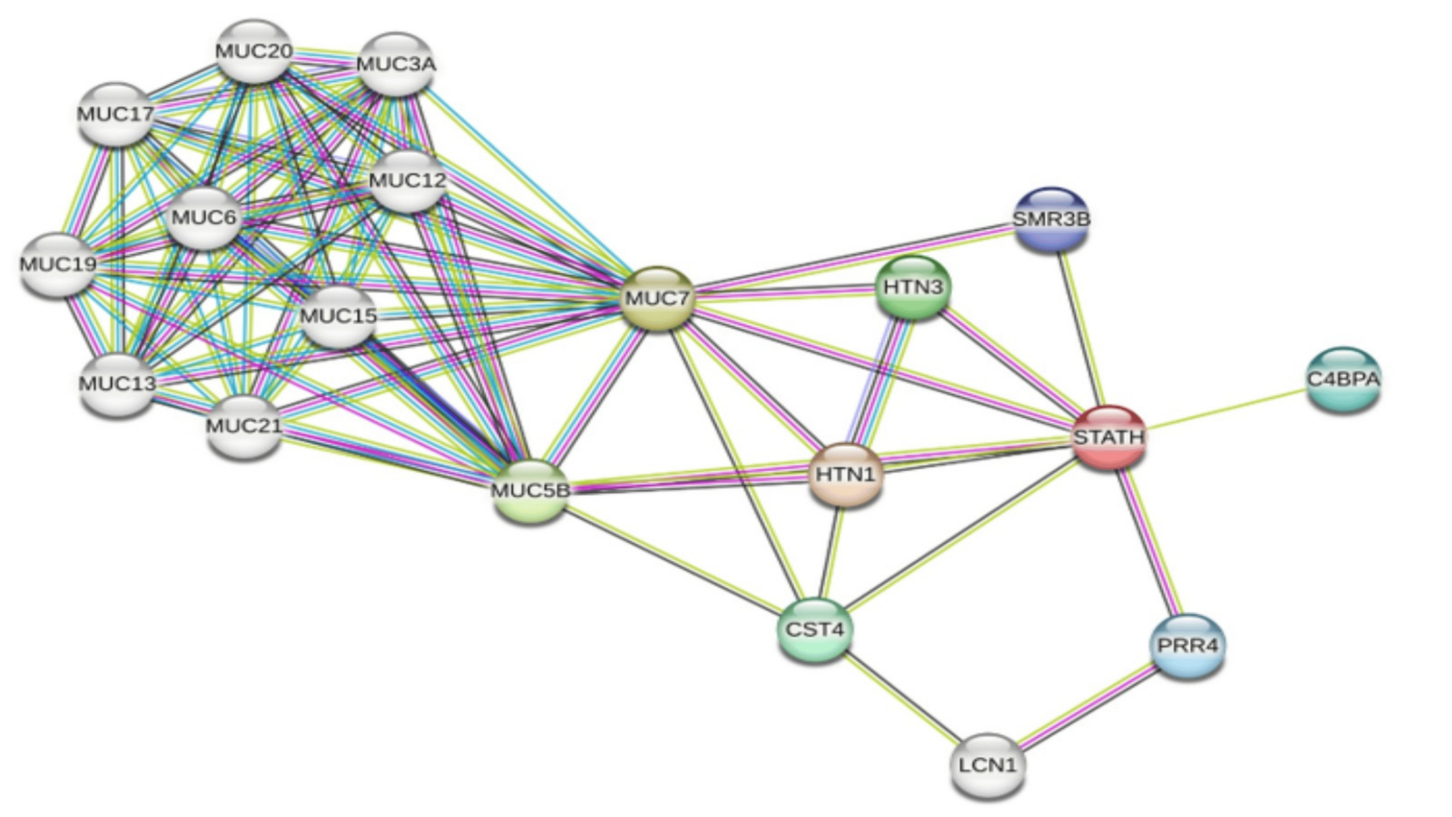
Protein-protein interaction (PPI) network of differentially expressed genes associated with STATH from the STRING database. Network nodes represent proteins, and edges represent protein-protein associations. STRING: Search Tool for the Retrieval of Interacting Genes/Proteins.

A PPI network was constructed via the STRING database to further investigate the functional role of STATH. To reveal the normal function of STATH, it was queried as a single protein, and the network included 19 proteins connected by 73 interactions, with an average of 7.68 interactions per protein and tightly connected local clustering (coefficient=0.784). The number of observed interactions was greater than expected by chance (expected=18), and the PPI enrichment (p<1.0e-16, p<0.001) confirmed that the connections were statistically significant. As shown in Figure 9, STATH strongly interacts with the salivary protein mucins, which are known contributors to lubrication and protection of the oral cavity. The strongest interaction was observed with MUC7 (degree=15), followed by MUC5B (degree=13). Other strongly interacting genes included MUC12, MUC13, MUC15, MUC17, MUC19, MUC20, MUC21, MUC3A, and MUC6 (all with degree=10), as well as STATH (degree=8), indicating that MUC7 functions as a hub gene in the constructed network and that STATH may play a key protective role in maintaining oral mucosal integrity.

**Figure 10:**
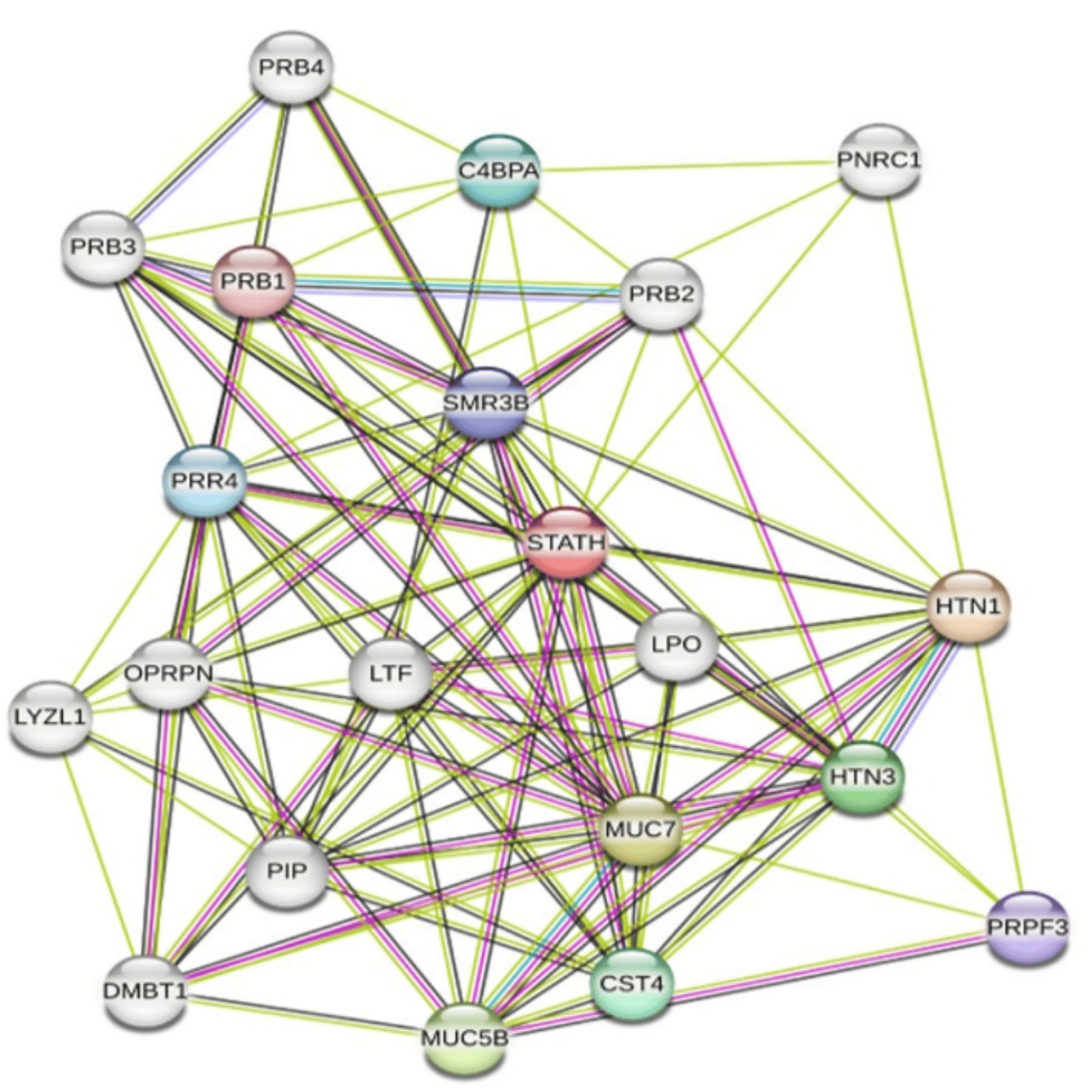
PPIs of the top 25 co-expressed genes associated with STATH from the STRING database. PPI: Protein-protein interaction; STRING: Search Tool for the Retrieval of Interacting Genes/Proteins.

To reveal its functional relevance in HNSCC, STATH was queried along with the top 25 positively co-expressed genes. As shown in Figure 10, the constructed network included 11 nodes and 36 edges, with an average node degree of 6.55 and average local clustering (coefficient of 0.826). The expected number of edges in a random network of the same size was 10, and the network showed significant PPI enrichment (p=2.63e-10, p<0.001). The criteria were relaxed to a medium confidence score of 0.4. The top interactor was MUC7 (degree=15), followed by PRR4 and HTN3 (degree=14), HTN1 (=13), and SMR3B and LTF (=12).

## Discussion

To our knowledge, this is the first study to investigate STATH as a specific and potential transcriptomic biomarker in HNSCC and to address its role in HNSCC tumorigenesis. We found that STATH is specifically downregulated in HNSCC, with expression significantly lower in tumor tissues compared with normal tissues, but not in other cancer types (Figure 1) and significantly varies by ethnicity, age, tumor grade, and nodal metastasis (Figure 2). STATH is generally hypermethylated, with a slight but significant decrease in tumors compared with normal controls (Figure 3), ruling out promoter methylation as the main mechanism underlying STATH downregulation in HNSCC. Genetic alterations are rare (<1%) and do not significantly impact survival, with gene amplification being the most common type of mutation, ruling out mutation as the mechanism of STATH downregulation (Figure 4). STATH co-expression analysis revealed significant enrichment in 10 GO terms (Table 1). Among these pathways, key pathways relevant to our hypothesis include the role of salivary proteins in periodontitis (OR=118.80, adjusted, p=0.007; genes: LYZ, LTF) and proteins involved in dental caries (OR=59.36, p=0.007; genes: LYZ, CCL28). The co-expressed genes contribute to biological processes, such as antimicrobial and antibacterial humoral response, defense against bacteria and defense against gram-positive bacteria. At the molecular level, endopeptidase inhibitor activity is a significant pathway. At the cellular level, they are localized to tertiary and specific granules. The constructed PPI network shows that STATH is part of a coordinated salivary defense network (Figure 9). In HPV-positive tumors, STATH is moderately positively correlated with B cells and CD8⁺ T cells and weakly positively correlated with dendritic cells (Figure 5). In HPV-negative tumors, STATH is weakly negatively correlated with neutrophils and dendritic cells (Figure 5B). Higher STATH expression was associated with a 9% lower risk of death and better overall survival (Figure 5C).

Overall, our findings align closely with the findings of previous studies investigating STATH at both the protein and gene levels. Previous proteomic studies have reported that statherin is a potential diagnostic biomarker in HNSCC, consistently reporting reduced salivary statherin levels in affected individuals [23-25]. For example, a study revealed that salivary statherin levels were significantly lower in the saliva of patients with precancerous and cancerous oral lesions than in those of healthy controls, whereas no significant changes were observed in patients with inflammatory or salivary gland disorders [23]. Together, these findings suggest that decreased STATH is linked to tumorigenesis, reflecting a mechanism by which HNSCC tumors reprogram the salivary microenvironment, which is consistent with the hallmark of cancer tumor microenvironment reprogramming. Our GO terms and pathway enrichment analyses further support this idea (Table 1) by revealing that STATH co-expressed genes are associated with oral diseases, specifically periodontitis, which is a known risk factor for HNSCC, and dental caries, which is frequently observed in these patients [3,4].

Previous studies have established a protective role of salivary statherin in the oral cavity, with lower levels observed in individuals with carious lesions than in caries-free individuals and higher levels in individuals with periodontitis compared to gingivitis, which is likely a compensatory protective response [9,26]. However, in HNSCC, the pattern is clearly different, with STATH consistently being downregulated. STATH regulates calcium homeostasis in oral epithelial cells, which is critical for maintaining desmosomes and the protective cornified envelope. Loss of STATH may compromise these structures and combined with tumor-driven alterations in calcium-dependent and antimicrobial pathways, it could create a salivary microenvironment prone to chronic inflammation, microbial imbalance, and tumor progression. Previous authors have raised this possibility in a letter to the editor [27]. The high connectivity to MUC7, PRR4, and HTN3 in our PPI network suggests that STATH functions within a broader salivary defense network and that its downregulation in HNSCC could disrupt this network, impairing oral homeostasis. We propose that the dental caries and periodontitis often observed in HNSCC patients are consequences of tumor-induced reprogramming, whereby networks that normally protect enamel integrity and oral tissues are disrupted. Complementing our study similar transcriptomic studies reported STATH and MUC7 are downregulated hub genes that regulate key pathways in oral squamous cell carcinoma (OSCC), a major subtype of HNSCC [28,29].

Consistent with previous studies, our results show that higher STATH expression in HPV-positive HNSCC tumors is correlated with increased infiltration of B cells, CD8+ T cells, and dendritic cells, reflecting a stronger antitumor immune response and likely contributing to the better overall survival observed in the HPV-positive group (HR=0.822, log-rank p=0.039), emphasizing the potential of STATH as a prognostic biomarker specifically in HPV-positive HNSCC patients [30]. Overall, previous findings and our findings complement one another, revealing the downregulation of Statherin at multiple biological layers during tumor progression.

Limitations 

This study is based on in silico analyses, which limits its direct clinical relevance. The lack of oral health status data, incomplete overall survival information in some databases, and inability to validate in a wet lab, such as through direct measurement of salivary STATH levels are important limitations we faced in this study.

## Conclusions

Our findings show that STATH downregulation is specific to HNSCC and may serve as a diagnostic biomarker. This downregulation reflects tumor-driven reprogramming of the salivary and oral microenvironments, where suppression of STATH creates a feedback loop that impairs host defenses and promotes tumor progression. The resulting disruption of oral homeostasis, evident in the poor oral health frequently observed in HNSCC patients, highlights the biological importance of STATH suppression in tumorigenesis. In terms of its prognostic significance, higher STATH expression in HPV-positive HNSCC is correlated with increased immune cell infiltration and better overall prognosis. Higher STATH expression, regardless of the HPV status, is generally correlated with better prognosis; however, this finding requires further validation.

We recommend that future studies address the limitations of this study and include clinical validation of salivary STATH levels, including proteomic analyses of statherin. We also propose that exploring therapeutic interventions aimed at restoring STATH expression levels in HNSCC patients may help improve oral health and disrupt the feedback loop that facilitates the progression of HNSCC.
